# Association between alanine aminotransferase-to-aspartate aminotransferase ratio and obstructive sleep apnea: A cross-sectional study of NHANES

**DOI:** 10.1097/MD.0000000000045995

**Published:** 2025-11-14

**Authors:** Yunzhou Zheng, Hao Xu

**Affiliations:** a Clinical Laboratory, Bethune International Peace Hospital, Shijiazhuang, China; b Department of Ultrasound Diagnosis, Bethune International Peace Hospital, Shijiazhuang, China.

**Keywords:** alanine aminotransferase, aspartate aminotransferase, biomarker, NHANES, obstructive sleep apnea

## Abstract

Obstructive sleep apnea (OSA) has been associated with abnormal liver enzyme levels. However, the association between the alanine aminotransferase-to-aspartate aminotransferase (ALT/AST) ratio and OSA remains understudied in large, nationally representative samples. This study examined the relationship between ALT/AST ratio and OSA among adults in the United States. We conducted a cross-sectional analysis of 7371 participants aged ≥20 years, pooling data from 4 National Health and Nutrition Examination Survey cycles (2005–2006, 2007–2008, 2015–2016, and 2017–2018). Weighted multivariate logistic regression was used to assess the relationship between the ALT/AST ratio and OSA, complemented by dose–response curve fitting, stratified subgroup analyses, and sensitivity analyses. Among 7371 participants (mean age 47.07 years; 48.50% male), 3672 reported OSA symptoms. A key finding was a significant inverted L-shaped nonlinear relationship between the ALT/AST ratio and OSA risk (*P* for nonlinearity = 0.021), with a threshold identified at a ratio of 1.08. Below this threshold, each unit increase in the ALT/AST ratio was associated with an 84% increase in the odds of OSA (odds ratio = 1.84, 95% confidence interval: 1.03–3.32, *P* = .041). Above 1.08, no significant association was observed. In a traditional quartile analysis, participants in the highest quartile of the ALT/AST ratio had 55% higher odds of OSA compared to those in the lowest quartile (odds ratio = 1.55, 95% confidence interval: 1.26–1.89, *P* < .001). The findings were robust across subgroup and sensitivity analyses. The ALT/AST ratio shows an association with self-reported OSA in cross-sectional data. These findings should be confirmed in longitudinal studies before considering clinical application. Additionally, prospective cohort or interventional studies are required to validate the observed threshold effect.

## 1. Introduction

Obstructive sleep apnea (OSA) is a prevalent sleep-disordered disease in which repetitive upper airway obstruction occurs during sleep, resulting in hypoxemia, sleep fragmentation, or daytime somnolence.^[1–3]^ This increasingly common disease is posing a significant public health challenge. Epidemiological studies indicate that approximately 1 billion people worldwide are affected by OSA, with prevalence rates increasing with age and being higher in males than in females.^[4,5]^ This condition is associated with numerous health outcomes, including elevated risks of obesity, cardiovascular disease, liver damage, metabolic disorders, and cognitive impairment.^[5,6]^ Despite its clinical importance, the pathophysiology of OSA is not fully elucidated, hence the need for novel biomarkers to aid in early diagnosis and risk stratification.

In contrast to the classical aspartate aminotransferase-to-alanine aminotransferase ratio (AST/ALT, or De Ritis ratio), the alanine aminotransferase-to-aspartate aminotransferase (ALT/AST) ratio has recently garnered attention as a potential marker for systemic metabolic dysfunction^[7,8]^ – a key pathophysiological feature of OSA. This premise is supported by reports linking the ALT/AST ratio to the presence of nonalcoholic fatty liver disease,^[9,10]^ a condition that shares common risk factors, such as obesity and insulin resistance, with OSA.^[11]^ We hypothesized that the ALT/AST ratio might serve as a more specific indicator reflecting the metabolic disturbances underlying OSA. Consequently, the primary objective of this study was to investigate the association between the ALT/AST ratio and the prevalence of OSA.

The National Health and Nutrition Examination Survey (NHANES) is an invaluable epidemiological research resource that includes a sizable sample that is nationally representative of the entire US population.^[12]^ This provides an opportunity to investigate the relationship between ALT/AST ratio and OSA. In this study, we explored the relationship between the ALT/AST ratio and prevalence of OSA using data from the NHANES. We hypothesized that the ALT/AST ratio may be associated with the prevalence of OSA.

By elucidating the association between the ALT/AST ratio and OSA, the present investigation could contribute to risk stratification for OSA and inform the development of targeted screening and intervention strategies.

## 2. Materials and methods

### 2.1. Data sources

The present analysis draws on NHANES data. NHANES is a research program designed to evaluate the health and nutritional status of the U.S. population. To ensure that the results are representative of the entire U.S. population, the National Center for Health Statistics (NCHS) uses a multi-stage, complex probability sampling design. All relevant details sourced from the official NHANES website (https://www.cdc.gov/nchs/nhanes/). The research protocol was authorized by the Research Ethics Review Board of the NCHS. The original research protocol is available on the Ethics Review Board website (https://www.cdc.gov/nchs/nhanes/about/erb.html). The study was formally approved by the ethical review committee under protocol numbers #2005-06, #2011-17, and #2018-01. Written informed consent was obtained from all participants upon registration. The present secondary analysis of de-identified data was deemed exempt from additional institutional review.

### 2.2. Study design and participants

**Figure 1.**
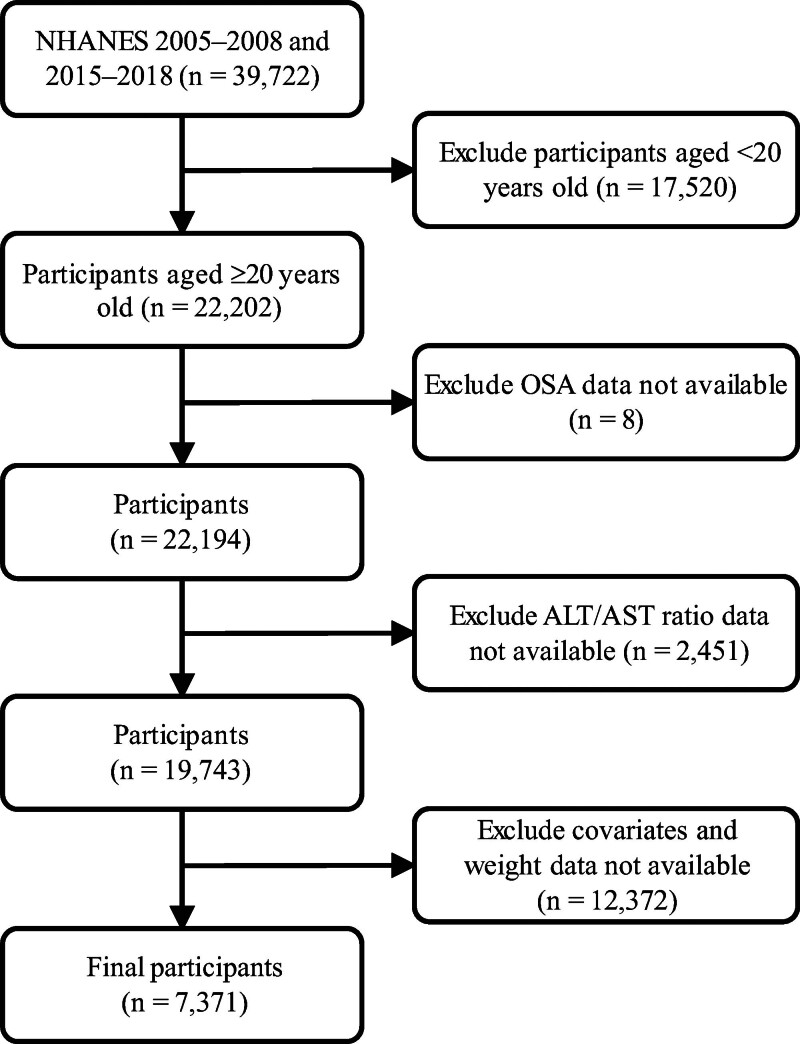
Flowchart of the study design. ALT/AST = alanine aminotransferase-to-aspartate aminotransferase, NHANES = National Health and Nutrition Examination Survey, OSA = obstructive sleep apnea.

This cross-sectional study was conducted in accordance with the Strengthening the Reporting of Observational Studies in Epidemiology (STROBE) guidelines. Data were obtained from 4 NHANES cycles (2005–2006, 2007–2008, 2015–2016, and 2017–2018). Data were analyzed between July and September 2024. The exclusion criteria for the study population were as follows: age <20 years, missing data on OSA or the ALT/AST ratio, and missing covariates. Ultimately, 7371 participants were enrolled in the study. A flowchart of the study design is shown in Figure 1.

### 2.3. Definition of ALT/AST ratio

The ALT/AST ratio was defined as the ratio of ALT to AST.

### 2.4. Definition of OSA

Based on previous research,^[13]^ OSA was ascertained based on affirmative responses to 3 relevant questions in the NHANES questionnaire: does snoring occur 3 or more times per week? Does the frequency of gasp, stopping breathing, or snort occur 3 or more times per week? Despite sleeping 7 or more hours per night, do you experience 16 to 30 instances of excessive daytime sleepiness per month? A “yes” response to any of these questions was considered diagnostic of OSA.

### 2.5. Covariates

Based on previous research^[14–18]^ and clinical judgment, the following covariates were selected: age, sex, race/ethnicity, education level, marital status, poverty income ratio, body mass index (BMI), smoking status, alcohol consumption, hypertension, diabetes, coronary heart disease (CHD), and laboratory variables. Race/ethnicity was classified into 4 categories: non-Hispanic White, non-Hispanic Black, Mexican American, and other race/ethnicity group. Education level: below the high school level, high school completion or equivalent, college-level education or higher. Marital status was classified into 3 mutually exclusive categories: never married; married or cohabiting; and widowed, divorced, or separated. BMI was calculated by dividing body weight (kg) by height squared (m). Smoking status was categorized based on lifetime cigarette consumption: individuals who had smoked < 100 cigarettes throughout their lifetime were classified as never smokers; those who had smoked ≥100 cigarettes but had quit were classified as former smokers; and those who had smoked ≥100 cigarettes and were still smoking were classified as current smokers.^[19]^ Consuming ≥12 alcoholic beverages within a 1-year period (or ever consuming any type of alcohol) was considered alcohol consumption, and individuals not meeting these criteria were classified as nondrinkers.^[20]^ Hypertension was assessed by self-reported participants.^[21]^ Participants who were informed of a diagnosis of hypertension during 2 or more separate clinical visits were classified as having hypertension. Those who were informed only once or not at all were classified as not having hypertension. Diabetes and CHD were identified through participant self-report.^[22,23]^ The laboratory variables included total protein, total bilirubin, albumin, alkaline phosphatase, gamma-glutamyl transferase, uric acid, blood urea nitrogen, creatinine, glucose, total cholesterol, triglycerides, and high-density lipoprotein cholesterol. All covariates were obtained from the NHANES database.

### 2.6. Statistical analysis

This study utilized publicly available data from the NHANES repository. Fasting weights were used in the weighted analyses. For each survey cycle, the sampling weight was set to 1/4 × WTSAF2YR (the fasting subsample 2-year MEC weight). Categorical variables were presented as unweighted counts (weighted percentages) and continuous variables as weighted means (standard error). For continuous variables, when data were normally distributed, group comparisons were performed using one-way ANOVA; when data were skewed, the Kruskal–Wallis test was employed. Categorical variables were compared using the χ² test. Multivariate logistic regression analysis was conducted to estimate the odds ratio (OR) and 95% confidence interval (CI) of the association between ALT/AST ratio and OSA. Multicollinearity was assessed (variance inflation factor <5). Missing covariates were handled using complete-case analysis (i.e., listwise deletion). As the analysis was based on a fixed, available dataset, prospective power calculation was not performed. However, the substantial sample size (n = 7371) yielded narrow CI for our primary associations (e.g., 95% CI: 1.15–1.89), which indicates precise effect estimates and affirms the adequacy of the sample size. Model 1 was adjusted for age, sex, race/ethnicity, education level, marital status, poverty income ratio, and BMI. Model 2 included additional adjustments for smoking status, alcohol consumption, hypertension, CHD, diabetes, and total protein, albumin, and total bilirubin levels. Model 3 was further adjusted for alkaline phosphatase, gamma-glutamyl transferase, blood urea nitrogen, creatinine, uric acid, glucose, high-density lipoprotein cholesterol, total cholesterol and triglycerides levels. The risk of overfitting in the multivariable model was considered low given the ample number of outcome events relative to the number of covariates. Restricted cubic spline regression (three knots placed at the 10th, 50th, and 90th percentiles) was employed to model the response association between the ALT/AST ratio and OSA, identify nonlinear relationships after adjusting for covariates in Model 3, and perform inflection point analysis.

Stratified analyses were performed across age (< 65 vs ≥65 years), sex, BMI (< 30 vs ≥30 kg/m²), alcohol consumption, and the presence of hypertension, CHD, or diabetes. Within each subgroup, all other covariates were adjusted to ascertain the specific effects of the stratified covariates on the association between ALT/AST ratio and OSA. Subgroup heterogeneity was evaluated using multivariable logistic regression, and interaction effects were assessed using the likelihood ratio test. To evaluate the robustness of our findings, we conducted 3 sensitivity analyses: excluding participants aged >70 years; excluding those with BMI ≥30 kg/m²; missing data in the covariates were handled using multiple imputation. All analyses were performed using a weighted approach and were conducted using R statistical software (version 4.1.2, https://www.r-project.org) and Free Statistics Software (version 2.0). Statistical significance was set at *P* <.05.

## 3. Results

### 3.1. Baseline characteristics

The ALT/AST ratio was used to allocate participants into 4 quartile-based groups. Table 1 presents the baseline patient characteristics. Overall, 49.19% of the participants reported OSA. Participants had a mean age of 47.07 (standard error = 0.37) years, and males accounting for 48.50% of the sample. The prevalence of OSA increased progressively across the ascending quartiles of the ALT/AST ratio, with rates of 37.53%, 43.42%, 52.33%, and 61.39% for Quartiles 1 through 4, respectively.

**Table 1:** Baseline characteristics according to the ALT/AST ratio quartiles.

Variables	ALT/AST ratio					
	Overall	Quartile 1 (0.21–0.77)	Quartile 2 (0.77–0.93)	Quartile 3 (0.93–1.13)	Quartile 4 (1.13–3.80)	*P*-value
Participants (n)	7371	1842	1824	1848	1857	–
Age (years), mean (SE)	47.07 (0.37)	48.61 (0.70)	48.18 (0.55)	47.31 (0.64)	44.53 (0.42)	<.001
Sex, male, n (%)	3613 (48.50)	618 (30.56)	738 (36.13)	965 (52.43)	1292 (71.27)	<.001
Race/Ethnicity, n (%)						
Non-Hispanic White	3224 (68.86)	812 (66.90)	842 (71.54)	789 (69.15)	781 (67.89)	<.001
Non-Hispanic Black	1490 (10.47)	490 (14.89)	393 (11.09)	372 (10.11)	235 (6.45)	
Mexican American	1230 (8.18)	205 (5.41)	260 (6.25)	339 (8.53)	426 (11.95)	
Other Race/Ethnicity	1427 (12.49)	335 (12.80)	329 (11.12)	348 (12.21)	415 (13.70)	
Education level, n (%)						
Less than high school	724 (4.87)	170 (4.99)	176 (4.61)	186 (4.36)	192 (5.49)	.029
High school or equivalent	2768 (34.60)	717 (36.44)	677 (34.19)	657 (31.40)	717 (36.45)	
College or above	3879 (60.52)	955 (58.57)	971 (61.21)	1005 (64.24)	948 (58.06)	
Marital status, n (%)						
Never married	1195 (16.91)	341 (19.18)	279 (15.61)	290 (16.57)	285 (16.43)	<.001
Married or Living with a partner	4551 (64.64)	978 (57.80)	1109 (63.94)	1197 (67.13)	1267 (68.77)	
Widowed or divorced or separated	1625 (18.46)	523 (23.02)	436 (20.45)	361 (16.30)	305 (14.79)	
Poverty income ratio, mean (SE)	3.07 (0.04)	2.92 (0.06)	3.06 (0.06)	3.16 (0.07)	3.12 (0.06)	<.001
BMI, (kg/m²), mean (SE)	29.14 (0.14)	26.56 (0.19)	28.02 (0.24)	29.60 (0.23)	31.90 (0.23)	<.001
Smoking status, n (%)						
Never	3969 (53.15)	992 (53.92)	1008 (53.32)	993 (53.88)	976 (51.66)	.135
Former	1934 (26.42)	443 (23.71)	487 (26.52)	492 (26.17)	512 (28.90)	
Current	1468 (20.43)	407 (22.37)	329 (20.16)	363 (19.95)	369 (19.45)	
Alcohol consumption, n (%)	5532 (80.03)	1314 (76.28)	1310 (77.95)	1421 (81.52)	1487 (83.69)	<.001
Hypertension, n (%)	2198 (25.46)	542 (23.28)	535 (24.07)	591 (27.32)	530 (26.80)	.101
Coronary heart disease, n (%)	317 (3.56)	97 (4.46)	76 (3.46)	83 (3.61)	61 (2.83)	.208
Diabetes, n (%)	998 (9.37)	185 (6.83)	220 (7.58)	289 (10.37)	304 (12.19)	<.001
Total protein (g/L), mean (SE)	71.00 (0.11)	71.04 (0.17)	70.67 (0.14)	70.86 (0.18)	71.39 (0.17)	<.001
Albumin (g/L), mean (SE)	42.02 (0.10)	41.79 (0.14)	41.89 (0.11)	41.88 (0.16)	42.45 (0.13)	<.001
Total bilirubin (µmol/L), mean (SE)	11.37 (0.11)	10.94 (0.16)	11.08 (0.19)	11.60 (0.22)	11.79 (0.20)	.002
Alkaline phosphatase (U/L), mean (SE)	69.96 (0.48)	69.30 (1.07)	67.25 (0.73)	70.02 (0.71)	72.88 (0.84)	<.001
Gamma-glutamyl transferase (U/L), mean (SE)	28.67 (0.58)	23.50 (2.00)	21.67 (0.62)	28.37 (0.85)	39.64 (1.03)	<.001
Blood urea nitrogen (mmol/L), mean (SE)	4.86 (0.04)	4.80 (0.08)	4.76 (0.06)	4.92 (0.06)	4.96 (0.06)	<.001
Creatinine (µmol/L), mean (SE)	77.56 (0.42)	77.05 (0.83)	75.88 (0.89)	78.43 (0.76)	78.66 (0.50)	<.001
Uric acid (µmol/L), mean (SE)	325.41 (1.33)	301.54 (2.65)	310.24 (2.61)	331.03 (2.83)	354.15 (2.13)	<.001
Glucose (mmol/L), mean (SE)	5.95 (0.03)	5.57 (0.03)	5.75 (0.04)	6.02 (0.06)	6.40 (0.05)	<.001
High-density lipoprotein cholesterol (mmol/L), mean (SE)	1.42 (0.01)	1.59 (0.02)	1.49 (0.01)	1.39 (0.01)	1.22 (0.01)	<.001
Total cholesterol (mmol/L), mean (SE)	4.99 (0.02)	4.92 (0.04)	4.98 (0.03)	4.98 (0.03)	5.08 (0.04)	.006
Triglycerides (mmol/L), mean (SE)	1.41 (0.02)	1.14 (0.03)	1.26 (0.03)	1.42 (0.03)	1.75 (0.05)	<.001
OSA, n (%)	3672 (49.19)	737 (37.53)	831 (43.42)	973 (52.33)	1131 (61.39)	<.001

Categorical variables were expressed as unweighted counts (weighted percentages) and continuous variables as weighted means (standard errors).

ALT/AST = alanine aminotransferase-to-aspartate aminotransferase, BMI = body mass index, OSA = obstructive sleep apnea.

### 3.2. Relationship between the ALT/AST ratio and OSA

**Figure 2.**
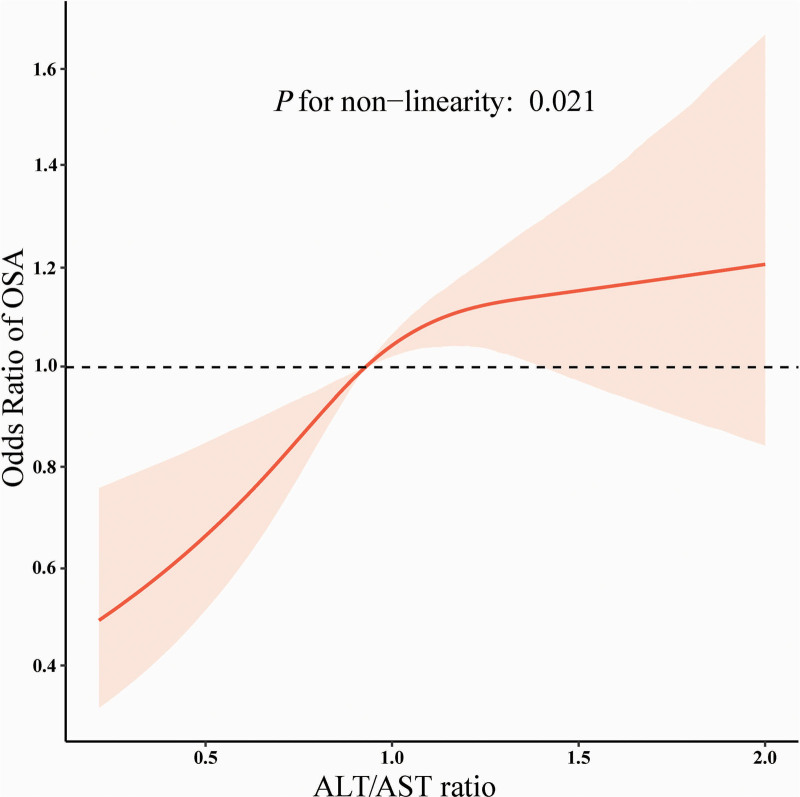
Weighted dose–response association of the ALT/AST ratio and obstructive sleep apnea. The solid orange line indicates odds ratio and the pink area indicates the 95% confidence interval. Adjusted for age, sex, race/ethnicity, education level, marital status, poverty income ratio, body mass index, smoking status, alcohol consumption, hypertension, coronary heart disease, diabetes, total protein, albumin, total bilirubin, alkaline phosphatase, gamma-glutamyl transferase, blood urea nitrogen, creatinine, uric acid, glucose, high-density lipoprotein cholesterol, total cholesterol, and triglycerides. Only 99% of the data are displayed. ALT/AST = alanine aminotransferase-to-aspartate aminotransferase, OSA = obstructive sleep apnea.

Table 2 presents the results of weighted multivariate logistic regression analysis. When the ALT/AST ratio was treated as a continuous variable, all models showed a positive association between ALT/AST ratio and OSA. In the unadjusted model, the OR was 2.84 (95% CI: 2.29–3.53). Following full adjustment in Model 3, the OR was 1.48 (95% CI: 1.15–1.89). The observed attenuation of the OR is likely due to the influence of demographic and metabolic confounding factors. This association persisted across the ALT/AST ratio quartiles. In Model 3, individuals in the highest quartile (Q4: 1.13–3.80) had an OR of 1.55 (95% CI 1.26–1.89; *P* < .001) compared with those in the lowest quartile (Q1: 0.21–0.77). In the restricted cubic spline model, the ALT/AST ratio and OSA showed an inverted L-shaped association (*P* = .021), with an inflection point at 1.08, as illustrated in Figure 2. Below an ALT/AST ratio of 1.08, OSA risk demonstrated a sharp increase with rising ratio levels. Above this threshold, the risk remained relatively stable. Two-segment regression indicated that among participants with an ALT/AST ratio < 1.08, each 1-unit increment was associated with an 84% higher OR for OSA (adjusted OR = 1.84; 95% CI 1.03–3.32; *P* = .041). However, no association was observed in participants with an ALT/AST ratio ≥ 1.08, as detailed in Table 3. Beyond this threshold, further elevation of the ALT/AST ratio was not associated with an additional risk of OSA. Thus, beyond the ALT/AST ratio of 1.08, additional increases in the ratio confer no further elevation in the OSA risk.

**Table 2:** Multivariable logistic regression of the ALT/AST ratio and obstructive sleep apnea.

Variables	n	Crude OR (95% CI)	*P*–value	Model 1 OR (95% CI)	*P*–value	Model 2 OR (95% CI)	*P*–value	Model 3 OR (95% CI)	*P*–value
ALT/AST ratio	7371	2.84 (2.29–3.53)	<.001	1.57 (1.22–2.02)	<.001	1.59 (1.24–2.05)	<.001	1.48 (1.15–1.89)	.003
ALT/AST ratio quartiles									
Q1 (0.21–0.77)	1842	1 (Ref)		1 (Ref)		1 (Ref)		1 (Ref)	
Q2 (0.77–0.93)	1824	1.28 (1.08–1.51)	.005	1.13 (0.96–1.32)	.140	1.14 (0.97–1.34)	.110	1.12 (0.95–1.32)	.170
Q3 (0.93–1.13)	1848	1.83 (1.53–2.18)	<.001	1.38 (1.14–1.67)	.001	1.39 (1.15–1.68)	.001	1.35 (1.11–1.64)	.004
Q4 (1.13–3.80)	1857	2.65 (2.19–3.19)	<.001	1.62 (1.32–1.99)	<.001	1.64 (1.34–2.00)	<.001	1.55 (1.26–1.89)	<.001
*P* for trend			<.001		<.001		<.001		<.001

Crude: unadjusted model.

Model 1: adjusted for age, sex, race/ethnicity, education level, marital status, poverty income ratio, and body mass index.

Model 2: adjusted for Model 1 and smoking status, alcohol consumption, hypertension, coronary heart disease, diabetes, total protein, albumin, and total bilirubin.

Model 3: adjusted for Model 2 and alkaline phosphatase, gamma-glutamyl transferase, blood urea nitrogen, creatinine, uric acid, glucose, high-density lipoprotein cholesterol, total cholesterol, and triglycerides.

The table shows the results of the multivariate regression analysis. All analyses were weighted to consider the complex sampling design of the survey and ensure that the results are representative of the studied population.

ALT/AST = alanine aminotransferase-to-aspartate aminotransferase, CI = confidence interval, OR = odds ratio, Q = quartile, Ref = reference.

**Table 3:** Threshold effect analysis of the ALT/AST ratio with the risk of obstructive sleep apnea.

ALT/AST ratio	Adjusted model	
	OR (95% CI)	*P*-value
<1.08	1.84 (1.03–3.32)	.041
≥1.08	0.93 (0.60–1.44)	.720

Adjusted for age, sex, race/ethnicity, education level, marital status, poverty income ratio, body mass index, smoking status, alcohol consumption, hypertension, coronary heart disease, diabetes, total protein, albumin, total bilirubin, alkaline phosphatase, gamma-glutamyl transferase, blood urea nitrogen, creatinine, uric acid, glucose, high-density lipoprotein cholesterol, total cholesterol, and triglycerides.

ALT/AST = alanine aminotransferase-to-aspartate aminotransferase, CI = confidence interval, OR = odds ratio.

### 3.3. Subgroup analyses of the association between the ALT/AST ratio and OSA

**Figure 3.**
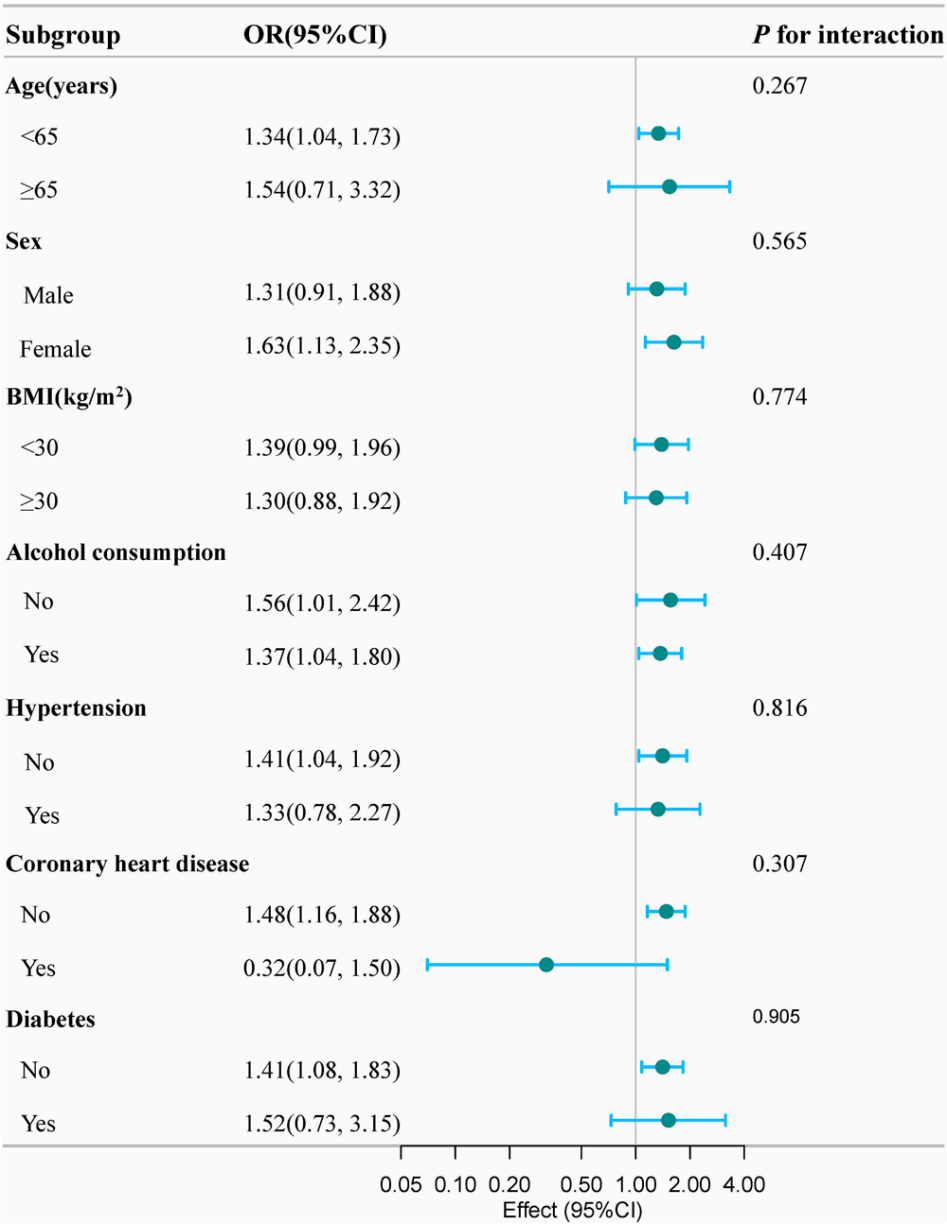
Subgroup analyses of the association between the ALT/AST ratio and obstructive sleep apnea. The stratifications were adjusted for all variables (age, sex, race/ethnicity, education level, marital status, poverty income ratio, body mass index, smoking status, alcohol consumption, hypertension, coronary heart disease, diabetes, total protein, albumin, total bilirubin, alkaline phosphatase, gamma-glutamyl transferase, blood urea nitrogen, creatinine, uric acid, glucose, high-density lipoprotein cholesterol, total cholesterol, and triglycerides), except for the stratification factor itself. Green dots indicate odds ratio and horizontal blue lines represent 95% confidence interval. ALT/AST = alanine aminotransferase-to-aspartate aminotransferase, BMI = body mass index, CI = confidence interval, OR = odds ratio.

To examine whether the association between the ALT/AST ratio and OSA was consistent across the different subgroups, we conducted stratified and interaction analyses (Fig. 3). Notably, the positive association between the ALT/AST ratio and OSA risk appeared to be more pronounced in females (OR, 1.63; 95% CI, 1.13–2.35) than in males (OR, 1.31; 95% CI, 0.91–1.88), although the test for interaction was not statistically significant (*P* for interaction = .565). Similarly, a stronger association was observed in participants without CHD (OR, 1.48; 95% CI, 1.16–1.88) compared to those with CHD (OR, 0.32; 95% CI, 0.07–1.50), suggesting a potential modifying effect of CHD status (*P* for interaction = 0.307). This finding warrants further investigation. The associations were generally consistent across other subgroups, including those defined by age, BMI, alcohol consumption, hypertension, and diabetes (All *P* for interaction >.05).

### 3.4. Sensitivity analysis

After excluding participants aged **>**70 years (Table 4), the OR for the association between the ALT/AST ratio and incident OSA was 1.41 (95% CI: 1.09–1.84, *P* = .012). Similarly, after removal of individuals with BMI ≥30 kg/m² (Table 5), the corresponding OR was 1.45 (95% CI: 1.03–2.04, *P* = .036). The effect sizes in these 2 sensitivity analyses were nearly identical to the primary result (OR, 1.48; 95% CI: 1.15–1.89; *P* = .003), further reinforcing the stability of the observed association. Following multiple imputation of covariates (Table 6), a weighted multivariable logistic regression analysis, adjusted for known confounders, yielded an OR of 1.52 (95% CI: 1.19–1.93, *P* = .001) for the association between the ALT/AST ratio and OSA risk. This result is consistent with the analysis using listwise deletion (OR: 1.48, 95% CI: 1.15–1.89, *P* = .003). These findings corroborate the robustness of the primary results.

**Table 4:** Sensitivity analysis in participants ≤70 years.

Variables	n	Crude OR (95% CI)	*P*-value	Model 4 OR (95% CI)	*P*-value
ALT/AST ratio	6259	2.89 (2.31–3.62)	<.001	1.41 (1.09–1.84)	.012
ALT/AST ratio quartiles					
Q1 (0.25–0.79)	1561	1 (Ref)		1 (Ref)	
Q2 (0.79–0.95)	1552	1.29 (1.05–1.57)	.014	1.07 (0.87–1.33)	.494
Q3 (0.95–1.16)	1579	1.92 (1.57–2.35)	<.001	1.32 (1.04–1.68)	.025
Q4 (1.16–3.80)	1567	2.85 (2.31–3.51)	<.001	1.58 (1.24–2.01)	<.001
*P* for trend	–	–	<.001	–	<.001

The table presents the results from a multivariate regression analysis of the ALT/AST ratio and obstructive sleep apnea in participants aged ≤70 years.

Model 4: Adjusted for age, sex, race/ethnicity, education level, marital status, poverty income ratio, body mass index, smoking status, alcohol consumption, hypertension, coronary heart disease, diabetes, total protein, albumin, total bilirubin, alkaline phosphatase, gamma-glutamyl transferase, blood urea nitrogen, creatinine, uric acid, glucose, high-density lipoprotein cholesterol, total cholesterol, and triglycerides

ALT/AST = alanine aminotransferase-to-aspartate aminotransferase ratio, CI = confidence interval, OR = odds ratio, Q = quartile, Ref = reference.

**Table 5:** Sensitivity analysis in participants with BMI < 30 kg/m².

Variables	n	Crude OR (95% CI)	*P*-value	Model 5 OR (95% CI)	*P*-value
ALT/AST ratio	4512	2.41 (1.79–3.24)	<.001	1.45 (1.03–2.04)	.036
ALT/AST ratio quartiles					
Q1 (0.21–0.74)	1111	1 (Ref)		1 (Ref)	
Q2 (0.74–0.88)	1144	1.14 (0.91–1.44)	.247	1.12 (0.88–1.44)	.352
Q3 (0.88–1.06)	1127	1.41 (1.11–1.78)	.005	1.20 (0.94–1.54)	.143
Q4 (1.07–3.80)	1130	2.22 (1.75–2.81)	<.001	1.58 (1.21–2.06)	.002
*P* for trend	–	–	<.001	–	.003

The table presents the results from a multivariate regression analysis of the ALT/AST ratio and obstructive sleep apnea in participants with BMI < 30 kg/m².

ALT/AST = alanine aminotransferase-to-aspartate aminotransferase ratio, CI = confidence interval, OR = odds ratio, Q = quartile, Ref = reference. Model 5: covariate adjustment matched Model 4 specifications.

**Table 6:** Sensitivity analysis following multiple imputation of covariates.

Variables	n	Crude OR (95%CI)	*P*-value	Model 6 OR (95%CI)	*P*-value
ALT/AST ratio	8773	2.84 (2.33–3.47)	<.001	1.52 (1.19–1.93)	.001
ALT/AST ratio quartiles					
Q1 (0.21–0.77)	2185	1 (Ref)		1 (Ref)	
Q2 (0.77–0.92)	2201	1.26 (1.08–1.46)	.003	1.10 (0.95–1.27)	.177
Q3 (0.93–1.13)	2181	1.78 (1.52–2.09)	<.001	1.33 (1.11–1.59)	.003
Q4 (1.13–3.80)	2206	2.61 (2.19–3.11)	<.001	1.56 (1.30–1.87)	<.001
*P* for trend	–	–	<.001	–	<.001

The table presents the results of the multivariate regression analysis of the association between the ALT/AST ratio and obstructive sleep apnea following multiple imputation of covariates.

ALT/AST = alanine aminotransferase-to-aspartate aminotransferase ratio, CI = confidence interval, OR = odds ratio, Q = quartile, Ref = reference. Model 6: Covariate adjustment matched Model 4 specifications.

## 4. Discussion

A large-scale cross-sectional analysis of U.S. adults revealed an inverted L-shaped curve relationship between the ALT/AST ratio and OSA, with an inflection point at 1.08. When the ALT/AST ratio was < 1.08, it was positively associated with OSA risk. There was no association between the ALT/AST ratio and OSA risk when the ALT/AST ratio was ≥1.08. The findings were robust across subgroup and sensitivity analyses.

A previous meta-analysis^[1]^ reported significant differences in the standardized mean levels of ALT and AST between 668 patients with OSA and 404 controls, with ALT and AST levels increasing by 13.3% and 4.4%, respectively. However, no prior study has examined the relationship between ALT/AST ratio and OSA. A systematic review by Jin^[24]^ evaluated 9 studies (2272 participants) from January 2007 to April 2017 and found that OSA was associated with elevated ALT levels but was not significantly associated with AST levels. Our study addresses this gap by using NHANES data, which provides a larger sample size and weighted analyses to investigate the ALT/AST ratio in relation to OSA. Consequently, our findings can be generalized to adults in the United States.

Our study observed a significant nonlinear positive association between the ALT/AST ratio and the risk of OSA. Although the underlying mechanisms remain under investigation, this association is biologically plausible. First, an elevated ALT/AST ratio is a well-established marker for nonalcoholic fatty liver disease, a condition intrinsically linked to hepatic and systemic insulin resistance. Insulin resistance, in turn, promotes the development and severity of OSA through multiple mechanisms: it can lead to altered ventilatory control, contribute to visceral adiposity which mechanically impedes upper airway patency, and potentially impair the neuromuscular control of the upper airway.^[25,26]^ Conversely, OSA itself may exacerbate liver injury and further elevate the ALT/AST ratio, creating a vicious cycle. The hallmark of OSA – chronic intermittent hypoxia – induces oxidative stress and systemic inflammation. This pro-inflammatory state can directly promote hepatic inflammation, accelerate the progression from simple steatosis to steatohepatitis (NASH), and thereby worsen hepatocellular injury.^[27–29]^ This pathway may represent a key biological mechanism underlying the association between the ALT/AST ratio and OSA risk. However, further prospective studies are warranted to validate this hypothesis.

This study has several strengths. First, the ALT/AST ratio served as the exposure variable, thus avoiding the limitations associated with the use of a single indicator. Second, the sample size was sufficiently large to provide a robust statistical power. Third, the use of NHANES weighted analysis ensured that the research results were representative of the general adult population in the US. However, this study had some limitations. First, the cross-sectional design precludes causal inference between the ALT/AST ratio and OSA. Therefore, prospective cohort studies are warranted to further investigate this association. Second, the sample was restricted to individuals aged ≥20 years, which may limit the generalizability of our findings to the younger population. Third, the definition of OSA relied on self-reported questionnaire data rather than objective polysomnography. This approach is susceptible to misclassification, likely underestimates prevalence and may have resulted in an under- or overestimation of the true association.^[13,19,22]^ Fourth, despite adjustment for a range of covariates, potential residual confounding may persist due to unmeasured factors such as neck circumference, waist-to-hip ratio, sleep duration, and continuous positive airway pressure treatment. Fifth, the lack of data on OSA treatment (e.g., continuous positive airway pressure use) is a limitation, as it may be an unmeasured confounder that influences liver enzyme levels.

## 5. Conclusion

The ALT/AST ratio shows an association with self-reported OSA in cross-sectional data. These findings should be confirmed in longitudinal studies before considering clinical application. Additionally, prospective cohort or interventional studies are required to validate the observed threshold effect.

## Acknowledgments

The authors would like to thank the NHANES participants and the National Center for Health Statistics. We express our gratitude to Dr Jie Liu from the Department of Vascular and Endovascular Surgery, Chinese PLA General Hospital for his valuable contributions, including the selected topic and study design consultation.

## Author contributions

**Conceptualization:** Yunzhou Zheng, Hao Xu.

**Data curation:** Yunzhou Zheng, Hao Xu.

**Formal analysis:** Yunzhou Zheng, Hao Xu.

**Funding acquisition:** Yunzhou Zheng, Hao Xu.

**Investigation:** Yunzhou Zheng.

**Methodology:** Yunzhou Zheng, Hao Xu.

**Project administration:** Yunzhou Zheng.

**Resources:** Yunzhou Zheng.

**Software:** Yunzhou Zheng.

**Supervision:** Yunzhou Zheng.

**Validation:** Yunzhou Zheng, Hao Xu.

**Visualization:** Yunzhou Zheng, Hao Xu.

**Writing – original draft:** Yunzhou Zheng, Hao Xu.

**Writing – review & editing:** Yunzhou Zheng, Hao Xu.
